# Advantages of deep learning with convolutional neural network in detecting disc displacement of the temporomandibular joint in magnetic resonance imaging

**DOI:** 10.1038/s41598-022-15231-5

**Published:** 2022-07-05

**Authors:** Yeon-Hee Lee, Jong Hyun Won, Seunghyeon Kim, Q.-Schick Auh, Yung-Kyun Noh

**Affiliations:** 1 Department of Orofacial Pain and Oral Medicine, Kyung Hee University Dental Hospital, #613 Hoegi-dong, Dongdaemun-gu, Seoul, 02447 South Korea; 2 Department of Computer Science, Hanyang University, Seoul, Korea; 3 Robotics Laboratory, Department of Mechanical and Aerospace Engineering, Seoul National University, Seoul, Korea; 4 School of Computational Sciences, Korea Institute for Advanced Study (KIAS), Seoul, 02455 Korea

**Keywords:** Computational biology and bioinformatics, Biomarkers, Diseases, Medical research, Pathogenesis, Risk factors, Signs and symptoms

## Abstract

This study investigated the usefulness of deep learning-based automatic detection of anterior disc displacement (ADD) from magnetic resonance imaging (MRI) of patients with temporomandibular joint disorder (TMD). Sagittal MRI images of 2520 TMJs were collected from 861 men and 399 women (average age 37.33 ± 18.83 years). A deep learning algorithm with a convolutional neural network was developed. Data augmentation and the Adam optimizer were applied to reduce the risk of overfitting the deep-learning model. The prediction performances were compared between the models and human experts based on areas under the curve (AUCs). The fine-tuning model showed excellent prediction performance (AUC = 0.8775) and acceptable accuracy (approximately 77%). Comparing the AUC values of the from-scratch (0.8269) and freeze models (0.5858) showed lower performances of the other models compared to the fine-tuning model. In Grad-CAM visualizations, the fine-tuning scheme focused more on the TMJ disc when judging ADD, and the sparsity was higher than that of the from-scratch scheme (84.69% vs. 55.61%, p < 0.05). The three fine-tuned ensemble models using different data augmentation techniques showed a prediction accuracy of 83%. Moreover, the AUC values of ADD were higher when patients with TMD were divided by age (0.8549–0.9275) and sex (male: 0.8483, female: 0.9276). While the accuracy of the ensemble model was higher than that of human experts, the difference was not significant (p = 0.1987–0.0671). Learning from pre-trained weights allowed the fine-tuning model to outperform the from-scratch model. Another benefit of the fine-tuning model for diagnosing ADD of TMJ in Grad-CAM analysis was the deactivation of unwanted gradient values to provide clearer visualizations compared to the from-scratch model. The Grad-CAM visualizations also agreed with the model learned through important features in the joint disc area. The accuracy was further improved by an ensemble of three fine-tuning models using diversified data. The main benefits of this model were the higher specificity compared to human experts, which may be useful for preventing true negative cases, and the maintenance of its prediction accuracy across sexes and ages, suggesting a generalized prediction.

## Introduction

Temporomandibular disorder (TMD) is an umbrella term for pain and dysfunction of the temporomandibular joint (TMJ) and masticatory muscles^1^. TMJ noise, limited mouth-opening, tinnitus, ear pain, neck and shoulder pain, and headaches may be accompanied by TMD pain in the TMJ and masticatory muscle areas. TMD is highly common, with 39% of the world’s population showing at least one sign or symptom of TMD and 25% having pain associated with TMD^2^. The prevalence of TMD in women is more than double that in men^3^. Unlike other joint diseases, the prevalence of which increase with age, TMD has a high prevalence in children and adolescents, often occurring in young people aged 20–45 years^4,5^. TMD has a multifactorial etiology including contributing factors such as physical, psychological, genetic, and hormonal factors. The common causes of TMD include microtrauma such as clenching and bruising, macrotrauma, mental challenges including anxiety and depression, sleep problems, and malnutrition^6^. The complex clinical features of TMD require a comprehensive approach.

The TMJ is one of the most complex joints in humans. The TMJ is bilateral and comprises the articular surfaces of the mandibular condyle and temporal bone. The TMJ discs between the bones, located between the superior and inferior joint spaces, have a high collagen content for durability and rigidity, which helps the mandible to rotate and translate and serves as a cushion for occlusal force^7^. TMJ displacement, also known as internal disc derangement, is an abnormal relationship among the articular disc, mandibular condyle, and mandibular fossa^8^. While the most frequent displacement of the disc is anterior to the mandibular condyle, it can also occur posteriorly^9^. Therefore, research has mainly been conducted on anterior disc displacement (ADD) of the TMJ. Uniquely, TMJ discs do not have direct nerve distribution or vascularization owing to their complex anatomic features. Alternatively, retrodiscal tissue, which is the posterior attachment of the TMJ disc, is characterized by various blood vessels and nerves that are critical for pathophysiological processes^7^. However, changes in the position or shape of the TMJ disc can further promote TMD development.

Individuals with TMJ disc displacement can be symptomatic or asymptomatic. TMJ disc displacement may only cause TMJ noises such as clicking or popping, whereas pain may cause limitations in mouth opening or restricted mandible movement^10^. A previous MRI study observed ADD in 33–41% of asymptomatic joints^11^. The stomatognathic system adapts to changes in the position of the TMJ disc and undergoes adaptive remodeling, resulting in a painless, asymptomatic state^9^. However, long-term, TMJ disc displacement ultimately leads to TMJ inflammatory response or progression to osteoarthritis. The condylar cartilage is vulnerable to damage caused by wear and tear over time. The bony cartilage of the TMJ disc is damaged and can no longer cushion. If not properly treated, subjective symptoms not only worsen but also adversely affect the surrounding tissue, ultimately progressing to osteoarthritis^7,12^. These pathological changes in disc displacement and articular cartilage are irreversible, chronic TMD, and may cause sociopsychological problems in patients with TMD.

MRI is considered the gold standard for evaluating the TMJ soft tissue and disc-condylar relationship, as well as determining disc displacement. The standard protocol for the MRI diagnosis of ADD uses the superior position of the condyle (12 o’clock position) as the reference point for the posterior band of the disc. Wilkes first proposed a commonly used classification of TMJ disc displacement^13^. This classification describes disc displacement and osseous changes in hard tissues, in which MRI is required to confirm these findings. Rammelsberg described an alternative technique for determining TMJ disc displacement using the functional anterior superior portion of the condyle as a reference position for a normal disc position^14^. However, the evaluation of an MR image is generally subjective and the interpretation can change depending on the interpreter’s experience and MR sequences. Observers can also make different diagnoses for the same patient, depending on the examination conditions and imaging modality. Thus, it is essential to establish a standardized MRI outcome for an appropriate diagnosis to ensure the diagnosis repeatability and reproducibility^15^. Additionally, MRI interpretations still fall short of showing a clear association with reported symptoms. Moreover, the correlations between clinical signs and symptoms and imaging findings in all TMD patient groups remain controversial^16^.

Machine learning is a subfield of artificial intelligence in which, instead of explicitly programming instructions, a machine learns to perform a task through mathematical analysis of a given set of data. Deep learning using convolutional neural networks (CNNs), a subclass of machine learning, is the most advanced artificial intelligence technology, and is increasingly used to automatically detect pathological features in medical images^17^. CNN algorithms must be trained using large amounts of annotated imaging data to develop predictive models to automatically detect specific pathological images^18^. Deep learning has several applications in dentistry. Our previous study automatically estimated age groups based on CNN and first molar images from panoramic radiography^19^. A previous study used the area under the curve (AUC) value to automatically detect TMJ osteoarthritis using cone-beam computed tomography (CBCT) was 0.86^20^. A CNN study of TMJ disc evaluation before orthodontic treatment reported an AUC ≥ 0.86^21^. However, no artificial intelligence models are yet available to automatically detect ADD in MR images of patients with TMD. We hypothesized that deep learning models with good data augmentation might outperform human clinicians in MRI readings when using the same data, which will benefit TMD diagnostics.

## Methods

The research protocol for this study was reviewed to ensure its compliance with the principles of the Declaration of Helsinki and approved by the Institutional Review Board of Kyung Hee University Dental Hospital in Seoul, South Korea (KHD IRB, IRB No-KH-DT21022). Informed consent was obtained from all participants.

### Study population

**Figure 1:**
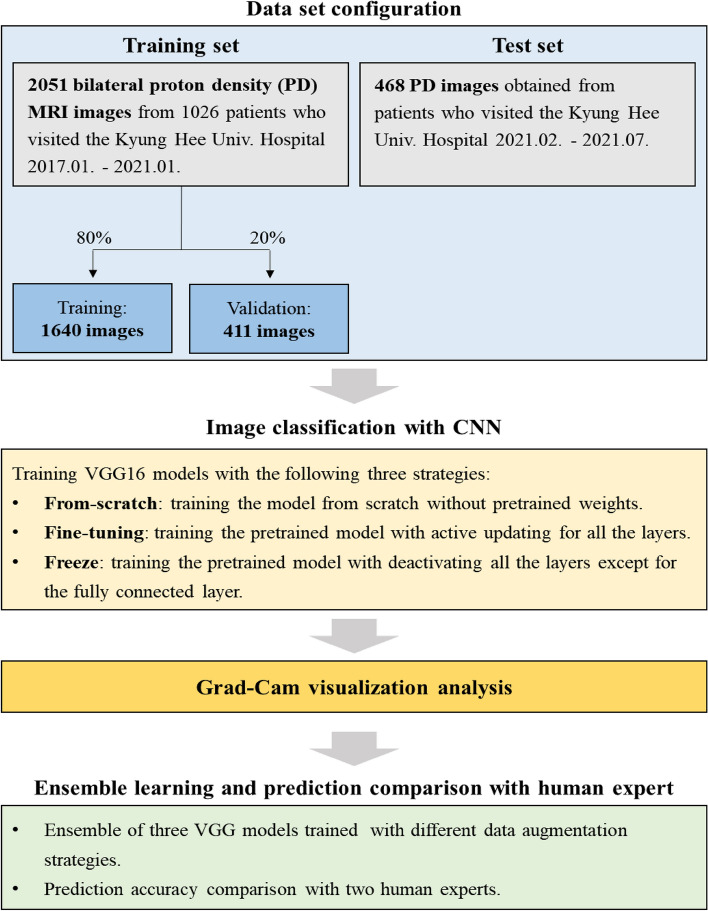
Study flowchart.

Figure 1 shows a flowchart of the present study. The study population comprised 1260 patients with TMD (861 men and 399 women, mean age = 37.33 ± 18.83 years), who visited Kyung Hee University Dental Hospital with TMD between January 2017 and July 2021. A TMD specialist with > 7 years of experience in TMD diagnosed TMD based on the criteria for TMD Axis I^22^.

The exclusion criteria were serious previous injuries such as unstable multiple traumas and facial fractures; systemic diseases potentially affecting the TMJ such as rheumatoid disease and systemic osteoarthritis; psychological problems; pregnancy; and psychiatric or neurological disorders. Cases in which the TMJ discs were not observed on MRI and where neither signal strength nor contour could define the structure as a TMJ disc were also excluded.

Among a total of 1260 patients (2520 TMJs), 2051 bilateral MRI images with proton density from 1026 patients (81.4%) who visited the hospital between January 2017 and January 2021 comprised the training set, while 468 images from 234 patients (18.6%) who visited the hospital between February 2021 and July 2021 comprised the evaluation dataset. When training the CNN models, 20% of the training set was used for training validation (Fig. 1).

### MRI image acquisition

All patients underwent MRI examinations of the bilateral TMJ. The MR images were obtained using a 3.0T MRI system (Genesis Signa; GE Medical System) with a 6 cm × 8 cm diameter surface coil. All scans involved sagittal oblique sections of ≤ 3 mm, a 15 cm field of view, and a 256 × 224 matrix. T2-weighted images (T2WIs) were obtained using a 2,650/82 TR/TE sequence; T1-weighted images (T1WIs) were obtained using a 650/14 TR/TE sequence; and proton density images were obtained using a 2,650/82 TR/TE sequence. Spin-echo sagittal MR images were obtained using an axial localizer.

### Accurate determination of TMJ disc displacement

**Figure 2:**
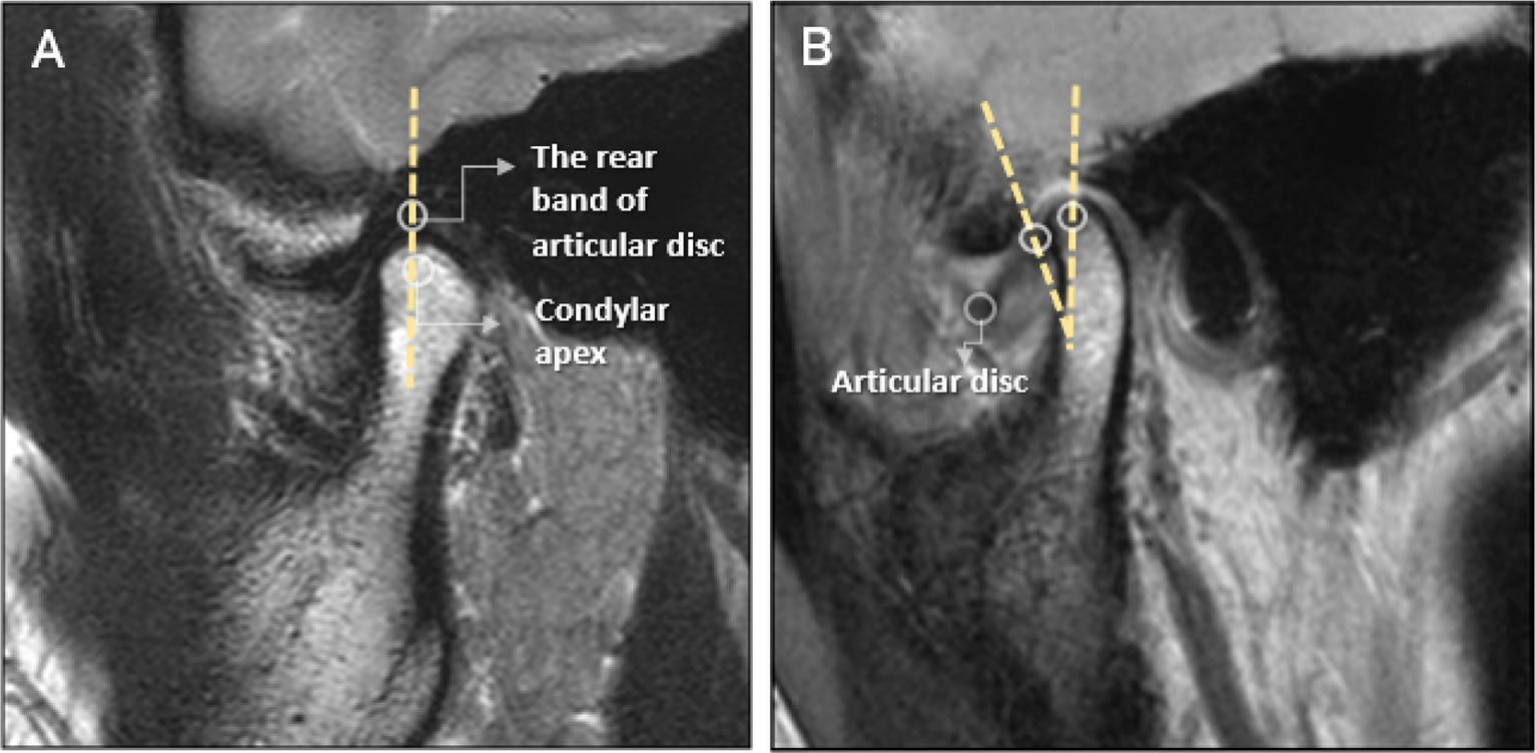
Magnetic resonance imaging (MRI) features of non-anterior disc displacement (non-ADD) (**A**) and ADD (**B**). Non-ADD: the rear band of the articular disc is located at 12 o’clock relative to the condylar apex at the closed position. ADD: the rear band of the articular disc moves forward beyond the normal range in the closed-mouth position.

The left and right sides of one patient with bilateral TMJs and ADD were assessed separately. The MRI image observation indicators for the TMJ in patients with TMD were^23^:

(i) Non-ADD: the rear band of the articular disc was located at the 12 o’clock position relative to the condylar apex in the closed position. The combination of the rear belt and double-plate area was located between 10 and 12 o’clock.
(ii) ADD: The back strap of the articular disc moved forward beyond the normal range in the closed-mouth position (Fig. 2).

The ADD was determined for the right and left sides of each patient. All T1-weighted images (T1WIs), T2WIs, and proton density images were referenced to determine the presence or absence of ADD to be learned by the deep learning model. All MRI investigations and interpretations were conducted by two investigators with > 7 years of experience in head and neck MRI. Internal consistency was represented using Cronbach’s α, and test–retest reliability was represented using intraclass correlation coefficient (ICC). The ICC was 0.91. Any disagreement in the MRI readings for ADD was resolved through discussion until a consensus was reached. Posterior disc displacement was not observed in this study.

### Interpretation of ADD with CNN models

As the performance of the CNN model was better when targeting proton density MR images compared to T2WIs and T1WIs, we used proton density MR images. The input MR images were pre-processed as follows: First, they were resized to 224 × 224 and then converted to three-channel images, with each channel having the same grayscale image. As a result, the dimensions of the inputs were set to 224 × 224 × 3.

The pre-trained three-dimensional VGG16 models were used for image classification. VGG16 is a CNN architecture that won the 2014 ILSVR competition and has been evaluated as one of the best vision model architectures to date. VGG16 succeeded in training a network that was twice as deep as the existing AlexNet 8-layers model, reducing the error rate by half^24^. VGG16 comprises a convolutional layer, three fully connected layers, a 3 × 3 convolutional filter, stride, padding 1, 2 × 2 max pooling, and a rectified linear unit (ReLU)^25^. We selected this model for its simple structure because our interest was not only to achieve high AUC scores but also to analyze the learned features and activation maps.

Three different machine learning schemes were tested. The first, “fine-tuning,” trained all layers of the pre-trained model from the very beginning. The second scheme, “from scratch,” trained a model without applying pre-trained weights. The last, “freeze,” trained the last layer of the pre-trained model only, preventing the training of the other layers. The evaluation metrics were the AUC and accuracy^26^. For the accuracy, specificity and sensitivity of the models were obtained from the optimal operating value earned by the Youden’s index calculated in the validation set^27^. All three schemes applied the same data augmentation techniques to 32 samples per batch. The fine-tuning and from-scratch models used a learning rate of 1e^-4^ with 15 and 30 epochs, respectively, while the freeze model used a rate of 5e^-4^ with 150 epochs. All three schemes used the Adam optimizer.

### Ensemble model with data augmentation

**Figure 3:**
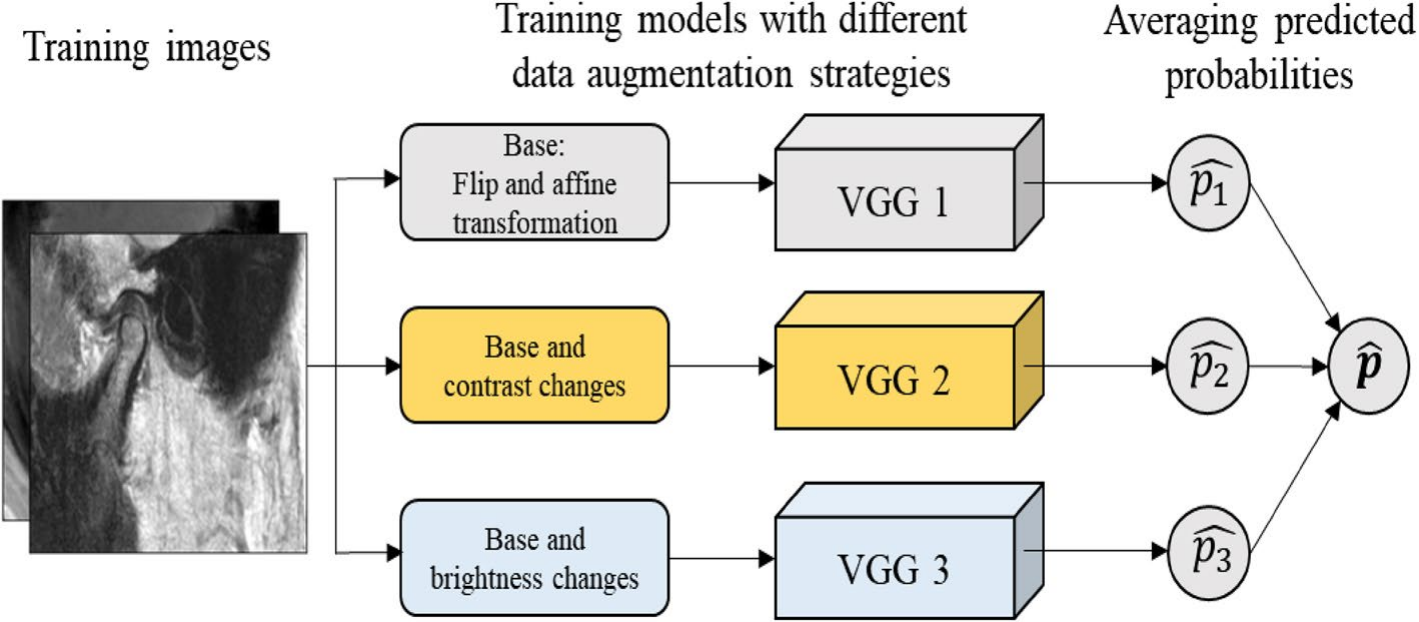
Architecture of the convolutional neural network (CNN) ensemble model. Three VGG16 models are learned using different data augmentation techniques. Base: randomly flip vertical and affine transform image. Contrast changes: randomly changed contrast values and applied histogram equalizations. Brightness changes: randomly changed image brightness. CNN, convolutional neural net.

An additional ensemble method was used to test the improvement in the prediction performance of the single fine-tuned model. Three different data augmentation techniques were applied to train the three fine-tuning models, and the predicted outputs were averaged (Fig. 3). This “data” ensemble was derived from the idea that using diversified data helps improve generalization performance more than applying a single CNN model^28^.

### Visual analysis to specify significant regions using Grad-CAM

For a deeper understanding of the learned features of the fine-tuning and from-scratch models, we analyzed their Grad-CAM images. We compared the same sample images that both models correctly predicted as positive images. Grad-CAM shows the most significant region for prediction by obtaining the importance weight ${\alpha }_{k}^{c}$, which is equal to the averaged value $\frac{\partial {y}^{c}}{\partial {A}^{k}}$ where ${y}^{c}$ is the logit of class $c$ and ${A}^{k}$ is the $k$-th activation map. The Grad-CAM heat map ${L}^{c}$ is then obtained as follows:

$${L}_{c}=ReLU\left({\sum }_{k}{a}_{k}^{c}{A}^{k}\right).$$

Because the heat map ${L}^{c}$ visualizes the significant pixels that change ${y}^{c}$ the most, applying the heat map to the input image shows the most important region for prediction. For visualization, we present some of the best Grad-CAM images.

### Validation of MRI findings by human experts

Finally, we compared the prediction results of the CNN models to those of two human experts who evaluated the same test set. Under the same conditions as the CNN models, given only proton density MR images of the TMJ of patients with TMD, the experts diagnosed non-ADD or ADD. The accuracy, specificity, and sensitivity were compared between CNN models and human experts. The human experts were blinded to each other and relied on their knowledge and experience in reading the MR images. Their ICC was 0.84.

### Statistical methods

Descriptive statistics are reported as means ± standard deviation or numbers with percentages, as appropriate. To analyze the distribution of discontinuous data, we used χ^2^ tests for equality of proportions, Fisher’s exact tests, and Bonferroni tests. All statistical analyses were performed using IBM SPSS Statistics for Windows, Version 22.0 (IBM Corp., Armonk, NY, USA), R Version 4.0.2 (R Foundation for Statistical Computing, Vienna, Austria), and Python Version 3.9.7 (Python Software Foundation, DE, USA). A receiver operating characteristic (ROC) curve was plotted and the AUC was calculated for each model, in which AUC = 0.5 indicated no discrimination, 0.6 ≥ AUC > 0.5 indicated poor discrimination, 0.7 ≥ AUC > 0.6 indicated acceptable discrimination, 0.8 ≥ AUC > 0.7 indicated excellent discrimination, and AUC > 0.9 indicated outstanding discrimination^29^. McNemar’s test was used to compare the prediction accuracies of the CNN models to those of the human experts. Statistical significance was set at a two-tailed p-value of < 0.05.

### Institutional review board

The research protocol was reviewed in compliance with the Helsinki Declaration and approved by the Institutional Review Board of Kyung Hee University Dental Hospital in Seoul, South Korea (IRB No-KH-DT21022). Informed consent was obtained from all participants.

### Informed consent

Informed consent was obtained from all the subjects involved in the study.

## Results

### Prediction results of the three learning schemes

**Figure 4:**
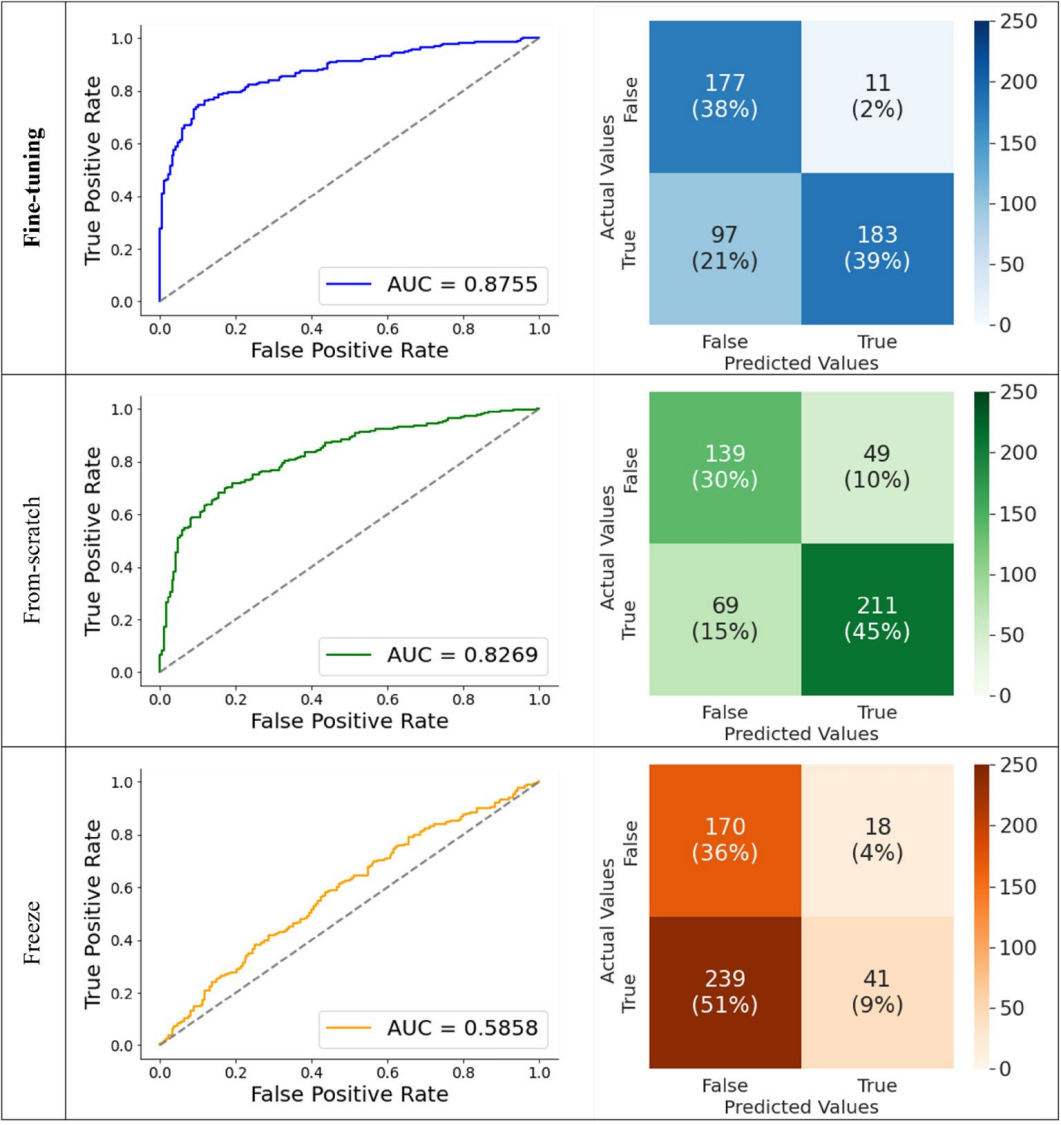
Classification results of three convolutional neural network (CNN) models using different learning strategies. Fine-tuning (blue), from-scratch (green), and freeze (orange). The fine-tuning model outperforms the others (area under the curve [AUC] = 0.8755).

Figure 4 shows the results of image classification using three different learning strategies. Each row represents a ROC curve with its AUC score and confusion matrix for the best classification of each model. The best prediction performance was observed in the fine-tuned model (AUC = 0.8755). The accuracy of this model when using the operating point in validation set was acceptable (approximately 77%). For this cutoff value, most errors were false negatives. The second-best model was from-scratch, which showed an AUC of approximately 0.83 and 75% accuracy. As this model was trained without pre-trained weights; that is, using only training image data itself, the high AUC confirmed that the discrimination of ADD from non-ADD was captured in the CNN features. However, a more accurate separation was obtained using pre-trained learning weights, boosting the AUC score by approximately 4%. The model trained using the freeze scheme exhibited the lowest AUC (AUC = 0.59).

### Grad-CAM visualization analysis of the fine-tuning and from-scratch models

**Figure 5:**
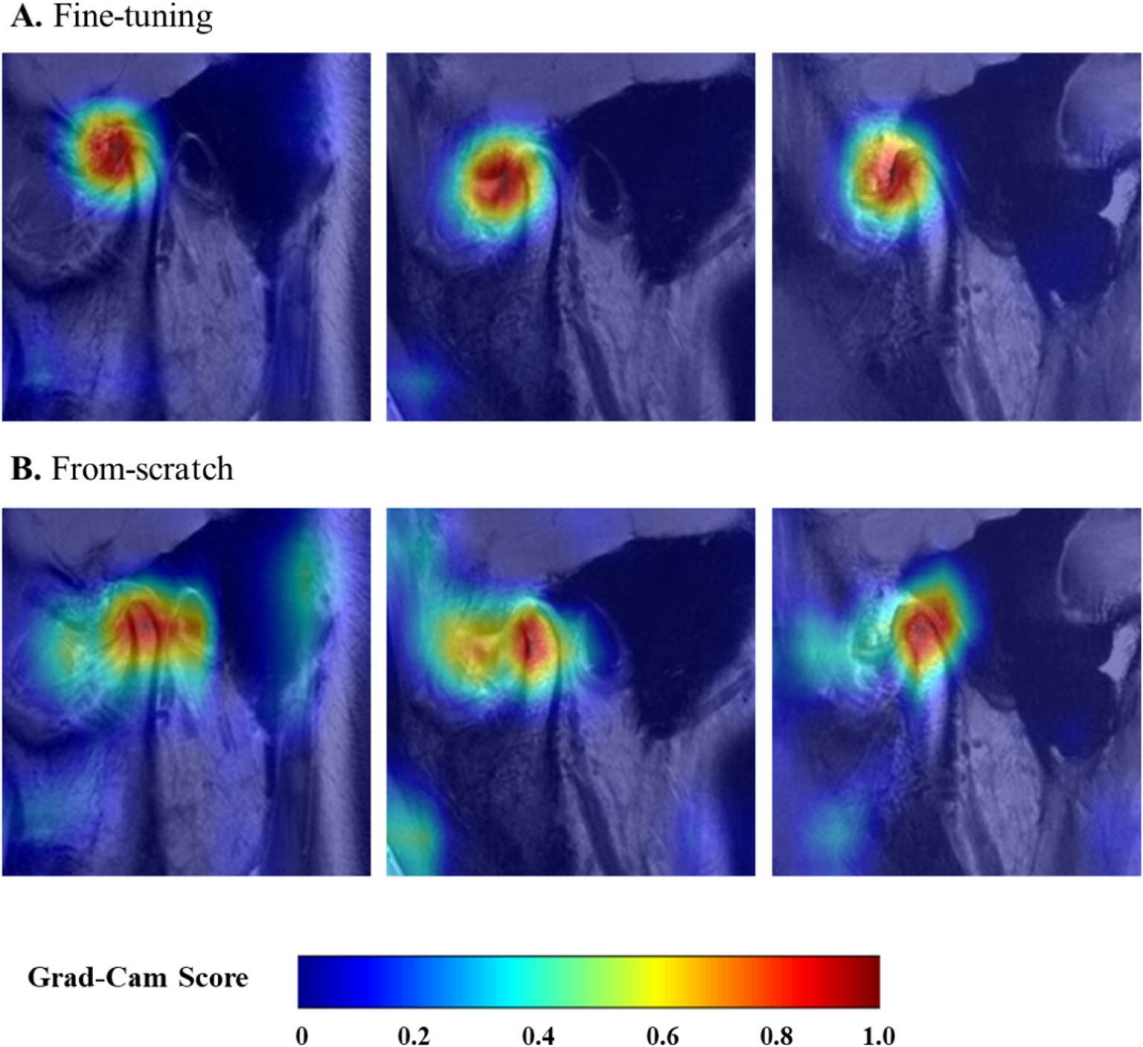
Grad-CAM visualizations of the fine-tuning (**A**) and from-scratch (**B**) models. The same sample images are displayed column-wise. The images from the fine-tuning model are more focused on the regions of interest (ROIs).

To understand and analyze the learned features of the fine-tuning and from-scratch models, we performed Grad-CAM analysis of the last channel using the test set images. Figure 5 shows the results of the three samples from the test set, where both models were correctly predicted as positive. Although the from-scratch model showed acceptable gradient scores according to the regions of interest (ROIs), the heatmaps of the fine-tuning model more consistently highlighted more concentrated areas of the TMJ ADD across the three images. Comparison of the fine-tuning model outputs showed that the from-scratch model tended to provide small gradient scores (0.2–0.4) to the uninteresting areas.

**Figure 6:**
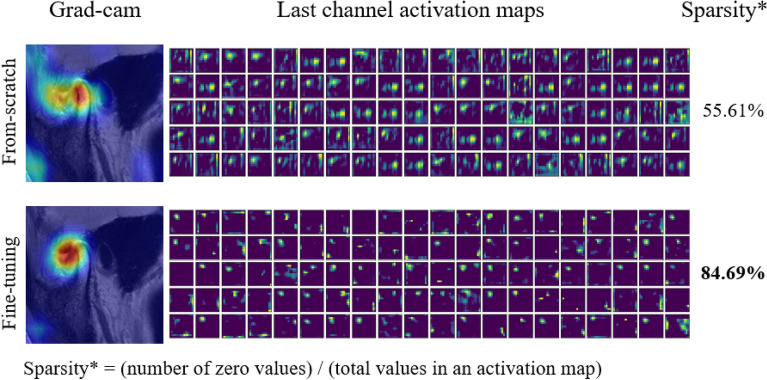
Last channel activation maps of both models using the same image. The first and second rows show the results of the from-scratch and fine-tuning models, respectively. Highly sparse activations are learned in the fine-tuning model, which provides a clearer Grad-CAM image.

Because the Grad-CAM feature of important weight ${\mathrm{\alpha }}_{c}^{k}$ was obtained by averaging the activation map ${A}^{k}$ element-wise, we further investigated the last channel activation maps of both models to understand the differences between their gradient heatmaps. We selected one image and used it to print 100 activation maps (out of 512) from the last channels of both models. The results are shown in Fig. 6. The first and second rows show the results of the from-scratch and fine-tuning models, respectively. Compared to the from-scratch model, the fine-tuning model learned a sparser representation in each activation map. When measuring the mean sparsity across all 512 maps, the zero area of each activation map of the fine-tuning model was $\approx$ 85% (using only $\approx$ 15% of the gradient signals). However, the sparsity of the from-scratch model was $\approx$ 56%, which was directly related to the gradient values of the uninteresting areas.

### Applying the ensemble method and prediction results

**Figure 7:**
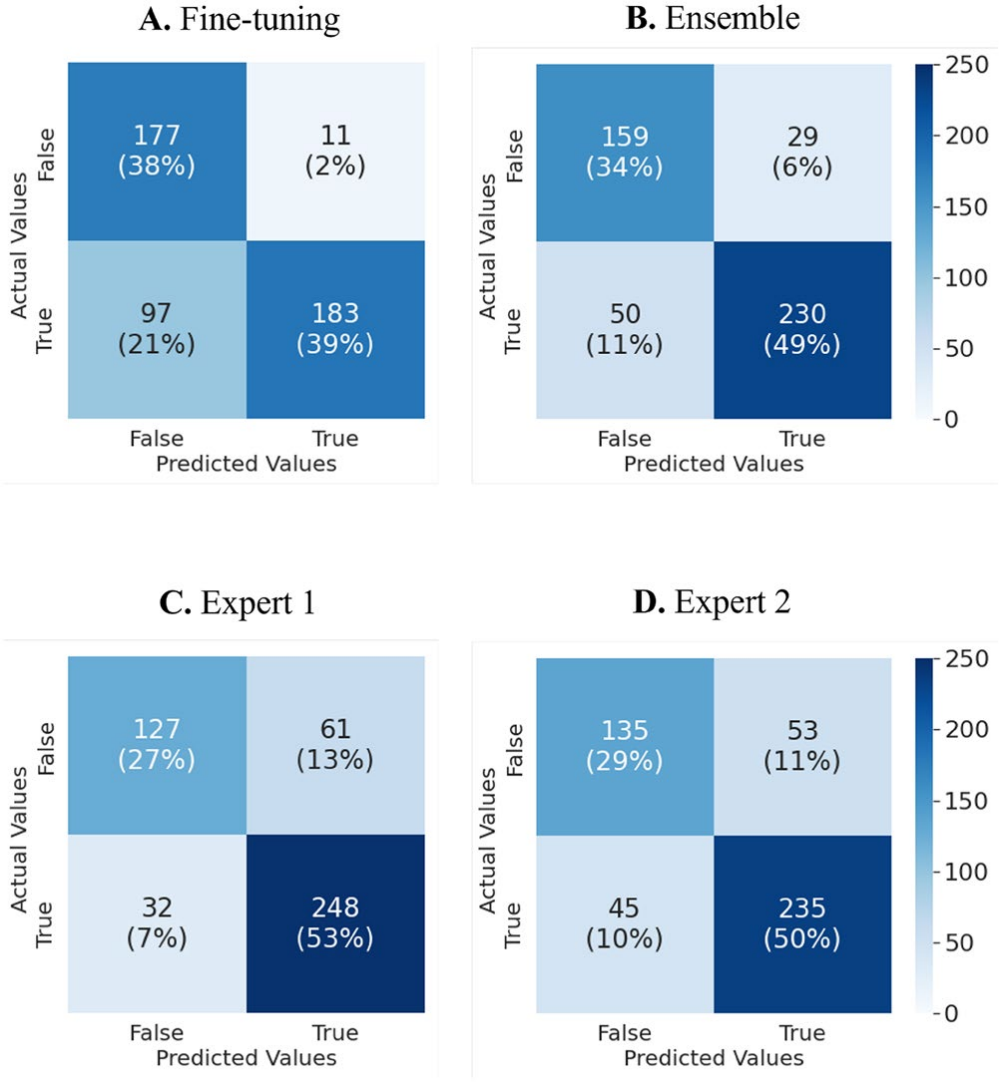
Confusion matrices of the single fine-tuning model (**A**), the ensemble model (**B**), and the two human experts (**C,D**).

An ensemble of three fine-tuning models using different data augmentation techniques was performed to examine how much accuracy would increase with a greater variety of data. The ensemble model receives three prediction probabilities and averages the probabilities to output the final predictions. We then compared the prediction results of the single fine-tuning model and ensemble model with those of two human experts. Table 1 summarizes the prediction results, and Fig. 7 shows the confusion matrices for each tester.

**Table 1:** Prediction comparisons of the single fine-tuning model, the ensemble model, and two human experts.

	Fine-tuning	Ensemble	Expert 1	Expert 2
Accuracy (%)	0.7692	**0.8312**	0.8013	0.7906
p-value (with Expert 1)	0.2211	0.1987	–	0.6445
p-value (with Expert 2)	0.3994	0.0671	0.6445	–
Sensitivity (%)	0.6536	0.8214	**0.8857**	0.8393
Specificity (%)	**0.9415**	0.8457	0.6755	0.7181

The case with the highest value was bolded.

Ensemble learning shows improved model accuracy to be slightly higher than that of two human experts. P-values: McNemar’s test using the results of the two experts as a reference.

First, the ensemble certainly improved the model’s prediction accuracy from 0.7692 to 0.8312. The sensitivity significantly improved from 65 to 82%. On the other hand, the specificity values were dropped from 94 to 85%. More importantly, the two models’ outputs did not differ statistically significantly from the experts’ discrimination results. Although the fine-tuning model had lower sensitivity than two experts, the test accuracy was not significantly different from that of the expert. However, regarding the specificity, the models showed a > 10% higher ability to reject undiagnosed patients compared to human experts. The operating points for the models were 0.8375 for the fine-tuning model and 0.7061 for the ensemble model, which was optimized from the validation set using Youden’s J statistics.

We further analyzed the model predictions by testing data subsets of sexes and various age spans (Table 2). We first confirmed that data from women were more accurately discriminated than data from men (AUC = 0.90 and 0.93 in women vs. 0.81 and 0.85 in men for the single model and the ensemble, respectively). When we separated the test set into four 20-year spans, the AUCs did not differ substantially among ages < 20, 21–40, and 41–60 years (AUCs $\approx$ 0.90). However, the AUC decreased slightly to 0.85 for age 61–83 years. Overall, the model’s discrimination competency maintained high AUCs (> 0.85 for the ensemble), which implied that learning in the CNN model was not gravely biased toward specific sex or age ranges.

**Table 2:** Detailed prediction results (AUCs) of test data sets according to sex and age groups.

		Fine-tuning model (AUC)	Ensemble model (AUC)
**Sex**			
Male	(n = 144)	0.8104	0.8483
Female	(n = 324)	0.8980	0.9276
**Age (years)**			
< 20	(n = 106)	0.8591	0.9040
21–40	(n = 142)	0.8918	0.9275
41–60	(n = 142)	0.8824	0.9059
over 61	(n = 66)	0.8145	0.8549

The ensemble model shows improved AUCs compared to one fine-tuning model. The AUC is substantially higher in women than in men. The AUCs do not differ significantly for ages 11 (minimum)–60 years, while the AUC for age > 61 years is decreased.

*AUC* area under the curve.

## Discussion

We examined the applicability of the CNN model for the automatic prediction of ADD and non-ADD and investigated the differences in prediction performance according to the age and sex of patients with TMD as well as the scheme of the CNN model. Recently, the deep learning computing paradigm has been regarded as the gold standard in machine learning in the artificial intelligence community, with CNNs the most utilized deep learning network type^30^. We first implemented three different learning schemes of CNN models to confirm (1) how much the pre-trained weights improved prediction performance, (2) whether there was a difference in prediction performance between humans and CNN models, and (3) whether the discrimination information of ADD from non-ADD could be learned through CNN features without the pre-trained weights. The fine-tuning model, trained with pre-trained weights, showed the best AUC (approximately 0.88); however, the from-scratch model also performed comparably well (AUC > 0.83), which confirmed our hypothesis that ADD information was successfully learned in CNN features without using pre-trained weights. The CNN model had a higher prediction specificity compared to the human experts. The ensemble of three fine-tuning models using different data perturbations also showed an improved accuracy, from 77 to 83%.

TMJ disc displacement is the most common cause of TMJ noise, restricted mandibular motion, and TMD progression^31^. Thus, accurate diagnosis is important. Joints with ADD with and without reduction were 2.73 and 8.25 times more likely to have osteoarthritis, respectively^32^. Additionally, complete ADD increased the risk of osteoarthritis by 10.88-fold^33^. Thus, disc displacement initially begins with symptoms such as clicking or popping in the TMJ area. If this problem is not resolved, there is a potential risk of TMD-related pain, mandibular function limitation, headaches, various psychological problems, and sleep problems^6,34^. The ADD prediction accuracies of the CNN models based on deep learning of artificial intelligence and trained human experts did not differ significantly. Although the sensitivity of the fine-tuning model was lower than that of the human experts, the ensemble model improved the sensitivity and achieved similar levels of accuracy with human experts. In terms of specificity, the CNN models were excellent, with a difference of 10% (94%, 85% vs. 68–72%). High specificity indicates a high probability of identifying a true negative^35^; this is desirable as the machines more accurately identify patients without ADD. The CNN model can make TMD diagnosis more efficient. Because the sensitivity for determining the presence or absence of ADD is higher in humans, the appropriate use of these machines by human experts could improve the diagnostic accuracy.

Among the three CNN schemes applied in this study, the fine-tuning model performed better than the scratch model. The difference between the two models was clear when Grad-CAM was applied to the final layer. The Grad-CAM-based color visualization approach is useful for unambiguously interpreting medical images^36^. The fine-tuning model is a state-of-the-art deep-learning method for disease detection from image data^37^. This model captured ADD regions more accurately and specifically compared to the from-scratch model, resulting in small gradient scores in unwanted areas. Accurately narrowing and recognizing the ROI is important in CNN models of deep learning^18,30,38^. We found that the fine-tuning model learned sparser activation maps, with approximately 85% empty areas for each map. In deep learning, increased sparsity implies that most of the weights are zero, with high sparsity potentially leading to increased space and time efficiency^39^. Only salient features remained when training from pre-trained weights while deactivating others^40^, which was directly connected to the localization quality of the Grad-CAM image. Although the from-scratch model performed comparably well (AUC = approximately 0.83), this result emphasizes the use of ImageNet pre-trained weights when applying CNN models in medical image diagnosis.

The tendency of the prediction performance of ADD to increase when the CNN model is applied according to sex and age group warrants further study. The prevalence of TMD is twice that in women compared to men^41^. We previously reported a significantly higher frequency of ADD among female patients with TMD with whiplash injury microtrauma compared to men^42^. Skeletally, the condylar volume did not differ significantly between men and women (691.26 ± 54.52 mm^3^ vs. 669.65 ± 58.80 mm^3^)^43^. Forensic dentistry showed that changes in the mandible and mandibular condyle are important for discriminating age and sex^44,45^. In the lumbar spine, decreased disc volume and disc degeneration are associated with increasing age^46^. However, unlike other joint problems, TMD is more common in children, adolescents, and young adults^5^. The differences by sex or age in the degeneration and displacement of the articular disc and ADD diagnosis accuracy remain unclear; furthermore, no quantitative in vivo MRI data are available on the factors influencing ADD diagnosis accuracy. The DC/TMD criteria, which are the most widely used worldwide for the diagnosis of TMD, indicate a 0.34–0.38 sensitivity of disc displacement with and without reduction without medical imaging^22^. The CNN model, a subgroup of artificial intelligence, automatically and more accurately detected ADD when MR image data are divided by age and sex and applied. Thus, ensemble model rather than single CNN models was a promising approach.

Similar studies using CNNs to predict TMJ disorders have been recently reported, which showed high AUCs for detecting TMJ areas^20,21^. Including our results, this means that CNN models may effectively distinguish between TMJs and non-TMJs. Unlike previous studies, this study reports new findings and addresses the possible benefits of using CNNs to detect TMJ disc displacement. First, using CNNs, the TMJ disc displacement can be discriminated without using pre-trained weights. However, it turned out that using pre-trained weights provides not only higher accuracy but also better quality of Grad-CAM visualizations by deactivating the unwanted gradients in activation maps. It is also notable that the ensemble model revealed its strength in detecting true-negative cases and showed consistent prediction accuracy across different sexes and various age groups.

### Limitations

Despite the high accuracy of the developed model, our study had some limitations. First, as the TMJs of patients with TMD were mostly diagnosed by ADD during data collection, the data labels were imbalanced toward positive samples by approximately 1:3 (23%:77%) between non-ADD and ADD, which might distract the freeze model from learning the discrimination. No specific strategy for class-imbalance in the training set was applied when training the CNN model. In fact, further experiments of the training model with weighted loss did not significantly improve the predictive performance (AUC was 0.87 for fine-tuning and 0.80 for from-scratch learning). Other strategies might boost the prediction scores and can be the future work. Second, this study was conducted using data from a single center, which may limit the model generalizability. To solve this problem, we diversified the data augmentation and developed several models; however, the possibility of overfitting the models remains. Moreover, the developed model could be used to evaluate only one sagittal MRI image for each prediction. However, the accurate diagnosis of ADD or TMD requires a comprehensive interpretation of all sagittal and coronal multilayer planes. Therefore, improving and validating the model performance requires the development of a deep learning algorithm to comprehensively recognize multiple layers of MR image data, in addition to a larger multicenter study.

## Conclusion

The results of this study illustrate the potential advantages of CNNs with pre-trained weights for the detection of TMJ disc displacement. Using pre-trained weights not only improved the prediction accuracy but also clarified Grad-CAM images by deactivating uninteresting gradient values. Grad-CAM visualization analysis confirmed that the most informative features were learned from the joint disc area. Moreover, even higher prediction accuracy was obtained from the CNN ensemble model using data perturbations. The model showed high specificity, which may aid human inspectors in reconsidering true negative diagnoses. Although the input MR images were from a single center and had a single sagittal plane, which restricts the use of comprehensive information, the model showed the potential generalization ability as it maintained a good performance across different sexes and ages.

## Data Availability

As these are patient data, any request for data disclosure will be discussed by the KHU-IRB before disclosure. The datasets generated and/or analyzed during the current study are not publicly available due to the protection of patient privacy according to the IRB permission but are available from the corresponding author on reasonable request.
